# Medical Library and Reading Room

**Published:** 1847-08

**Authors:** 


ARTICLE III.

### MEDICAL LIBRARY AND READING ROOM.

The Editors of this Journal and the faculty of Rush Medical College have established a reading room for the use of the profession and medical students. It is located in Chicago, opposite the Post Office, on Clark Street. All the principal medical journals published in this country, and a good library may be found there. Physicians and students from abroad are invited to call at this reading room, which will, at all times, be open to them without charge. D. B.

